# Crystal Structure of the SH3 Domain of ASAP1 in Complex with the Proline Rich Motif (PRM) of MICAL1 Reveals a Unique SH3/PRM Interaction Mode

**DOI:** 10.3390/ijms24021414

**Published:** 2023-01-11

**Authors:** Xuanyan Jia, Leishu Lin, Shun Xu, Lingxuan Li, Zhiyi Wei, Cong Yu, Fengfeng Niu

**Affiliations:** 1 School of Life Science and Technology, Harbin Institute of Technology, Harbin 150001, China; 2 Brain Research Center and Department of Biology, School of Life Sciences, Southern University of Science and Technology, Shenzhen 518055, China; 3 Guangdong Provincial Key Laboratory of Cell Microenvironment and Disease Research, Shenzhen Key Laboratory of Cell Microenvironment, Shenzhen 518055, China

**Keywords:** SH3, proline-rich motif, MICAL1, ASAP1, high affinity, protein–protein interaction, crystal structure

## Abstract

SH3 domains are common protein binding modules. The target sequence of SH3 domains is usually a proline-rich motif (PRM) containing a minimal “PxxP” sequence. The mechanism of how different SH3 domains specifically choose their targets from vast PxxP-containing sequences is still not very clear, as many reported SH3/PRM interactions are weak and promiscuous. Here, we identified the binding of the SH3 domain of ASAP1 to the PRM of MICAL1 with a sub-μM binding affinity, and determined the crystal structure of ASAP1-SH3 and MICAL1-PRM complex. Our structural and biochemical analyses revealed that the target-binding pocket of ASAP1-SH3 contains two negatively charged patches to recognize the “xPx + Px+” sequence in MICAL1-PRM and consequently strengthen the interaction, differing from the typical SH3/PRM interaction. This unique PRM-binding pocket is also found in the SH3 domains of GTPase Regulator associated with focal adhesion kinase (GRAF) and Src kinase associated phosphoprotein 1 (SKAP1), which we named SH3^AGS^. In addition, we searched the Swiss-Prot database and found ~130 proteins with the SH3^AGS^-binding PRM in silico. Finally, gene ontology analysis suggests that the strong interaction between the SH3*^AGS^*-containing proteins and their targets may play roles in actin cytoskeleton regulation and vesicle trafficking.

## 1. Introduction

Protein–protein interactions (PPIs) are universal and fundamental for cell activities. They employ many protein domains as the modules to mediate and construct the PPI network [1,2]. Src homology 3 (SH3) domain is one of the earliest discovered and widely studied PPI modules, which can recognize a diverse array of the proline-rich motifs (PRMs) [3]. Until now, about 300 SH3-containing proteins were identified in humans. They have been reported to play essential roles in various cell processes such as signal transduction, endocytosis, and actin cytoskeleton remodeling [4,5]. Interestingly, although varying in amino acid sequences, the SH3 domains have a conserved architecture containing five antiparallel β-strands (β1–β5) and three defined loops of RT-loop, n-Src-loop, and Distal-loop, as well as a short helix termed as 3_10_, which form a highly conserved hydrophobic groove to recognize PRMs [6]. Correspondingly, most PRMs are comprised of canonical PxxP-cored patterns (where x indicates any residue) with a polyproline type-II helical conformation [6]. However, the consensus PxxP sequence is insufficient to determine the PRM binding pose on SH3 domains. The PRMs contain a positively charged residue in the flanking sequence at either terminus of the PxxP sequence, which binds to a negatively charged cleft near the hydrophobic groove of SH3 domains. Based on the position of this positively charged residue, the canonical PRMs are grouped into class-I (+xxPxxP) and class-II (xPxxPx+) (where x indicates any residue and + indicates the positively charged residue R or K) [7,8].

In the recognition of SH3 to the class-II PRM (x_−1_P_0_x_1_x_2_P_3_x_4+5_, where the subscript number defines the position for each residue), the two conserved prolines at positions 0 and 3 provide the binding framework while the positively charged residue at position 5 provides the binding specificity [7,8]. In addition, the sequence analysis suggested that position 2 of the class-II PRM prefers a hydrophobic residue (xPxΦPx+, where Φ indicates the hydrophobic residue) [6]. Most of the bindings between SH3 domains and the class-II PRMs are detected to have relatively low affinities with a wide range of *K*_d_ ~20–200 μM [7,8,9,10,11,12,13,14,15,16,17,18,19]. 

Here, we reported a novel high-affinity binding between the PRM of MICAL1 (MICAL1-PRM) and the SH3 domain of ASAP1 (ASAP1-SH3), which shows an atypical Px + P-cored motif recognizing by a unique “Cxx(D/E)” sequence in the RT-loop of the SH3 domain. Based on structural, biochemical, and sequence analyses, we demonstrated the unique high-affinity binding mode between the SH3 domains in the ASAP/GRAF/SKAP family proteins and ~130 “Px + P”-containing targets. The gene ontology analysis indicates that these interactions may play roles in the actin cytoskeleton organization and vesicle trafficking. 

## 2. Results

### 2.1. ASAP1 Is a Novel Binding Partner of MICAL1 Mediated by the High-Affinity SH3/PRM Interaction 

**Figure 1:**
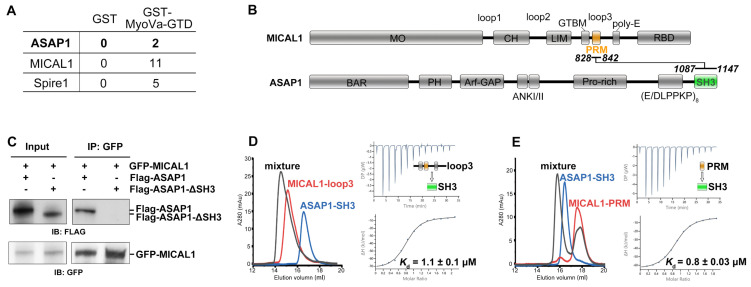
Identification of the MICAL1 and ASAP1 interaction mediated by the SH3/PRM recognition. (**A**) Peptide counts of ASAP1, MICAL1 and Spire1 binding to GST or GST-MyoVa-GTD by mass spectrum detection. (**B**) Domain organization of MICAL1 and ASAP1. The potential interacting regions of MICAL1-PRM and ASAP1-SH3 are highlighted. (**C**) The co-IP result showing the association of MICAL1 to ASAP1, but not ASAP1-ΔSH3, which is derived from Figure S1B. (**D**,**E**) aSEC and ITC investigations on the binding of ASAP1-SH3 to MICAL1-loop3 (**D**) and MICAL1-PRM (**E**), respectively.

Previously, we identified MICAL1, an F-actin disassembly factor, as the binding partner of Myosin Va (MyoVa) by using the globular tail domain (GTD) of MyoVa as the bait in the pulldown experiment coupled with mass spectrum analysis [20]. Besides MICAL1, another potential MyoVa-binding protein, Arf-GAP with SH3 domain, ANK repeat and PH domain-containing protein 1 (ASAP1), was also fished out (Figure 1A). ASAP1 was reported to play essential roles in both membrane trafficking (e.g., Rab8/11-coated vesicles) and actin cytoskeleton remodeling [21,22,23], which are highly associated with the MyoVa’s functions [24]. However, we failed to observe the interaction between MyoVa-GTD and ASAP1 by using co-IP assay (Figure S1A), implying that a third protein may bridge the interaction through its binding to both MyoVa-GTD and ASAP1. As MICAL1 and Spires are well-characterized GTD-binding proteins [20,25], we tested whether ASAP1 can interact with MICAL1 or Spire1. Consequently, compared to the faint band in the pull-down of Spire1 (Figure S1B), MICAL1 was detected to have an obvious association with ASAP1 (Figure S1B), suggesting that MICAL1 links MyoVa and ASAP1 together to form a ternary complex.

To understand how ASAP1 recognizes MICAL1, we mapped the interacting regions in these two proteins. As ASAP1 contains a C-terminal SH3 domain and MICAL1 contains a PRM in the third loop region (loop3) of MICAL1 (Figure 1B) [26,27], we suspected that ASAP1 may bind MICAL1 through its SH3 domain. Consistent with our hypothesis, the deletion of the SH3 domain from ASAP1 (ASAP1-ΔSH3) completely abolishes the binding of ASAP1 to MICAL1 (Figure 1C and Figure S1B). We further purified the two MICAL1 fragments containing the PRM sequence and the SH3 domain of ASAP1, respectively, and found that both MICAL1 fragments form the stable complex with ASAP1-SH3 (Figure 1D,E), confirming that ASAP1 binds to MICAL1 via the SH3/PRM interaction. Importantly, compared with the typical SH3/PRM interactions with ~20–200 μM binding affinity (Figure S2) [7,8,9,10,11,12,13,14,15,16,17,18,19], the isothermal titration calorimetry (ITC)-based analyses indicate that the interaction between ASAP1-SH3 and MICAL1-PRM has a relatively high affinity of ~1 μM (Figure 1E). The strong interaction between MICAL and ASAP1 not only explains why ASAP1 could be found in the pull-down experiments using MyoVa-GTD, but also suggests a unique binding of ASAP1-SH3 to MICAL1-PRM.

### 2.2. A Unique Cxx(D/E) Sequence in ASAP1-SH3 and K832 in the PxxP Motif of MICAL1-PRM Are Required for the High-Affinity Binding

**Figure 2:**
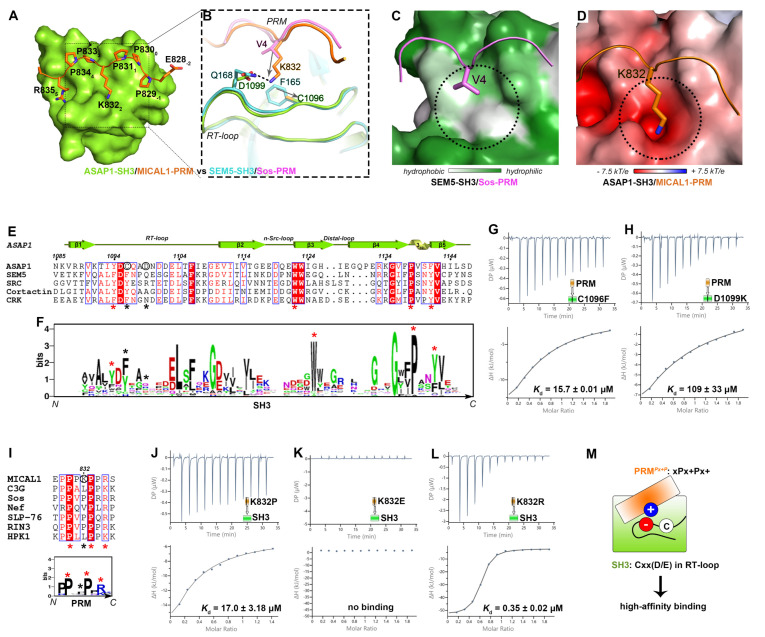
A Cxx(D/E) sequence in the RT-loop of ASAP1-SH3 determines the high-affinity binding to the PRM*^Px+P^* in MICAL1. (**A**) Complex structure of ASAP1-SH3 (green in surface mode) and MICAL1-PRM (orange in stick mode). The subscript number (−2 to 5) indicates the position of each residue in MICAL1-PRM. (**B**) Structural comparison showing the major interface difference between ASAP1-SH3/MICAL1-PRM and SEM5-SH3/Sos-PRM (PDB ID: 1SEM) complexes. The salt bridge is indicated by the black dash line. The length change of the sidechain of the residues in the corresponding position is indicated by gray arrows, respectively. (**C**,**D**) Surface analyses showing the hydrophobic pocket in SEM5-SH3 (**C**) and the negatively charged pocket in ASAP1-SH3 (**D**), respectively. (**E**,**F**) Sequence alignment (**E**) and Weblogo-style representation (**F**) of the SH3 domain sequences. The Weblogo-style representation was generated from the sequence analysis of >100 human SH3 domains. The conserved four aromatic residues in the hydrophobic groove of SH3 domains are labeled with red asterisks. The unique residues C1096 and D1099 in ASAP1 are circled and their corresponding positions are labeled with black asterisks. (**G**,**H**) ITC-based affinity measurements of MICAL1-PRM binding to the ASAP1-SH3 mutations. (**I**) Sequence alignment and Weblogo-style representation of the class-II PRMs. The conserved residues are labeled by red asterisks. The unique residue K832 in MICAL1 is circled and its corresponding position is labeled by a black asterisk. (**J**–**L**) ITC-based affinity measurements of MICAL1-PRM with the mutations of K832 binding to ASAP1-SH3. (**M**) A cartoon model summarizing the high-affinity binding between the SH3 with a unique Cxx(D/E) sequence in RT-loop and the PRM*^Px+P^*.

To unveil the molecular basis of such a high-affinity interaction between ASAP1 and MICAL1, using a shorter boundary (828–836) of MICAL1-PRM, we determined the crystal structure of ASAP1-SH3 and MICAL1-PRM complex at an atomic resolution of 1.17 Å with high quality (Figure 2A and Figure S3A,B and Table S1). In the complex structure, ASAP1-SH3 adopts a typical SH3 fold to interact with the two crucial prolines of the PxxP core in MICAL1-PRM (Figure 2A and Figure S3C). In addition to the proline binding, the positively charged residue R835 (position 5) at the C-terminus of MICAL1-PRM packs with the negatively charged patch on ASAP1-SH3 (Figure S3D), indicating that MICAL1-PRM belongs to the class-II PRM [6]. Interestingly, ASAP1-SH3 is highly conserved among the ASAP family (Figure S3E) while such a class-II PRM only exists in MICAL1 but not in MICAL2 and MICAL3 (Figure S3F). Consistent with the interface analysis, the interface mutations in ASAP1-SH3, like F1141Q and D1102K that disrupt the hydrophobic groove and negatively charged patch of ASAP1-SH3, respectively, abolish the binding of ASAP1 to MICAL1 (Figure S3G,H). Likewise, the charge-reverse mutation R835E in MICAL1-PRM eliminates the SH3/PRM interaction (Figure S3I).

Next, we carefully compared our structure to the SEM5-SH3/Sos-PRM complex structure (Figure 2B), which is regarded as the typical model to understand how SH3 recognizes the class-II PRM [8]. The two SH3 structures are similar to each other, including the hydrophobic groove and the negatively charged patch for sequestering two prolines and the C-terminal arginine in the PRMs, respectively (Figure S3C,D) [8]. However, the PRM-binding surface on the RT-loop of SH3 is significantly different between these two SH3 domains (Figure 2B). In the SEM5-SH3 structure, an aromatic residue F165 is located at the center of the hydrophobic pocket and provides hydrophobic contact with a valine (V4) in Sos-PRM (Figure 2B,C), whereas in ASAP1-SH3, this position is replaced by a cysteine, C1096, which has a short sidechain and thereby unlikely contacts with a short-sidechain residue in position 2 of PRMs (e.g., V4 in Sos-PRM) (Figure 2B). Instead, such a phenylalanine-to-cystine replacement allows the long sidechain of K832 in MICAL1-PRM to fit into this pocket of ASAP1-SH3 (Figure 2B,D). In addition, the positively charged tip of the sidechain of K832 is fixed by the interaction with negatively charged D1099 in ASAP1-SH3, corresponding to a glutamine residue (Q168) in SEM5-SH3 (Figure 2B,D). 

The above structural comparison suggests that the subtle changes at the two positions of the RT-loops in the SH3 domains between ASAP1 and SEM5 provide the differential binding to the PRMs. Compared with the unique residue C1096 in the RT-loop of ASAP1-SH3, the corresponding residue in most of SH3 domains is an aromatic residue, like phenylalanine (F) or tyrosine (Y) (Figure 2E,F) [6]. Either mutation C1096F or D1099K in ASAP1-SH3 dramatically decreases the affinity for MICAL1-PRM binding to a level comparable to those of the typical SH3/PRM interactions (Figure 2G,H). On the other hand, compared with the typical class-II PRMs, position 2 of MICAL1-PRM is a lysine (K832) rather than a hydrophobic residue (Figure 2I). Consistently, mutating K832 in MICAL1-PRM to a hydrophobic residue (K832P) weakens the binding affinity dramatically (Figure 2J). In contrast to the K832E mutant that completely loses the binding to ASAP1-SH3 (Figure 2K), the K832R mutation mildly enhances the ASAP1/MICAL1 interaction (Figure 2L), confirming the requirement of the positively charged residue for the high-affinity recognition. Notably, despite having a lysine (K1586) at position 2, APC-PRM was reported to interact with ASAP1-SH3 with a *K*_d_ of ~24 μM [28] (Figure S4A). By structural and sequence comparison of APC-PRM with MICAL1-PRM, we found that the second consensus proline at position 3 was substituted by a serine in APC-PRM (Figure S4A,B), which impairs the hydrophobic interaction and thereby reduces the binding of APC-PRM to ASAP1-SH3. 

Together, our structural, sequence and biochemical analyses demonstrated that ASAP1-SH3 contains a unique Cxx(D/E) sequence in the RT-loop, to create an additional negatively charged pocket in the PRM-binding groove for the high-affinity recognition to the MICAL1-PRM. This correspondingly contains an atypical class-II PRM with the xPx + Px+ pattern, termed as PRM*^Px+P^* (Figure 2M).

### 2.3. Identification of the Similar High-Affinity Binding Mode between SH3^AGS^ and PRM^Px+P^

**Figure 3:**
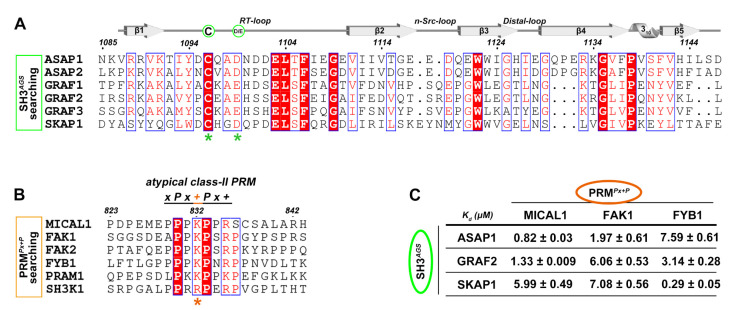
The SH3*^AGS^* recognition to their targets with the high-affinity binding. (**A**) Sequence alignment of the SH3 domains of ASAP, GRAF and SKAP proteins showing their identical Cxx(D/E) sequence in RT-loop. The unique cystine and negatively charged residue are labeled by green circles and asterisks. (**B**) Sequence alignment of the PRM-containing proteins targeted by ASAP, GRAF and SKAP proteins indicating an atypical class-II PRM motif. The unique positively charged residue is highlighted in orange and labeled by orange asterisk. (**C**) ITC-based affinity measurements of between the SH3*^AGS^* domains and their PRM*^Px+P^* targets.

It is intriguing to know whether the similar PRM-binding mode found in ASAP1-SH3 also occurs in other SH3 domains. We searched the SH3-containing proteins against the protein domain database [29] with the “Cxx(D/E)” sequence in the RT-loop. Six SH3-containing proteins were identified, including ASAP1/2, GRAF1/2/3 and SKAP1 (Figure 3A). Since these six proteins belong to the ASAP, GRAF, and SKAP families, we classified this group of SH3 domains as SH3*^AGS^*. Meanwhile, after the motif scanning of the PRM*^Px+P^* sequence pattern in the reported binding partners of SH3*^AGS^*, we found that many binding partners contain a lysine in position 2, such as FAK1 and FYB1, which are recognized by ASAP1 [30], GRAF2 [31] and SKAP1 [32], respectively (Figure 3B). Interestingly, MICAL1 has recently been identified to be recruited by GRAF2 for cell tubular structure formation [33], suggesting that these SH3*^AGS^*-containing proteins share a similar high-affinity binding mode to their targets, having the “xPx + Px+” sequence pattern. 

To confirm this unique recognition, we next measured the binding affinities between SH3*^AGS^* of ASAP1, GRAF2, SKAP1 and the PRM*^Px+P^* from MICAL1, FAK1, FYB1. Expectedly, the binding affinities of all pairs are in a range of ~0.3–8 μM (Figure 3C and Figure S5), stronger than those in the typical SH3/PRM interactions (Figure S2). Thus, with the guidance of the structure-based motif analysis and searching, a novel class of the SH3*^AGS^* recognition to their PRM*^Px+P^* targets with high affinity were identified. 

### 2.4. Functional Implications of the High-Affinity SH3^AGS^/PRM^Px+P^ Interaction 

**Figure 4:**
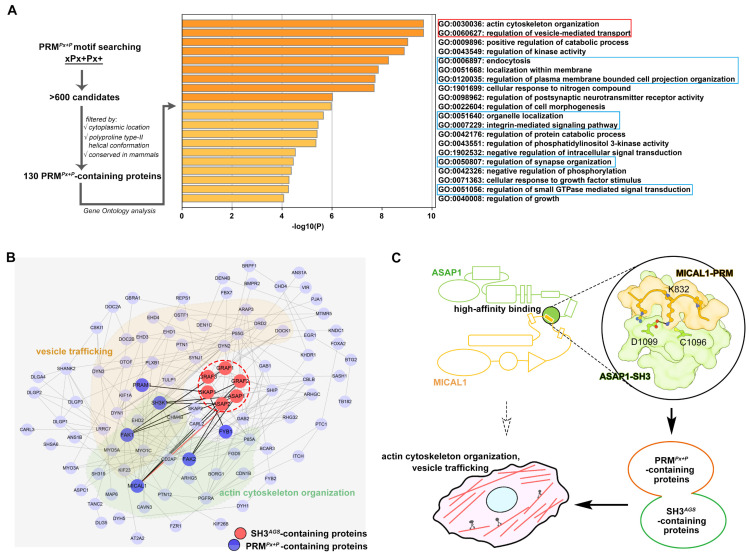
Functional implications of the high-affinity SH3*^AGS^*/PRM*^Px+P^* recognitions. (**A**) Motif-searching to identify the PRM*^Px+P^*-containing proteins and the following Gene Ontology analysis. Two major functions were boxed in red, while other cellular membrane/cytoskeleton-related fields are boxed in blue. (**B**) A protein–protein interaction network among the SH3^AGS^-containing ASAP/GRAF/SKAP proteins (red) and the PRM*^Px+P^*-containing proteins (blue). The known SH3*^AGS^* binding partners were highlighted in dark blue. The targets involving vesicle trafficking and actin cytoskeleton organization are classified with a different color background. (**C**) A flow chart summarizing the identification of the high-affinity SH3*^AGS^*/PRM*^Px+P^* recognitions and their potential physiological roles.

Compared to the typical SH3/PRM interaction, the SH3*^AGS^*/PRM*^Px+P^* interaction shows an unusual high-affinity interaction to enhance the binding specificity. To investigate the functional role of the SH3*^AGS^*/PRM*^Px+P^* interaction, we further analyzed more than 600 PRM*^Px+P^*-containing candidates that were generated by searching the “xPx + Px+” sequence pattern against the Swiss-Prot database in the Scansite web server [34]. After filtering with different conditions, including the cytoplasmic location, the polyproline-II helical conformation predicted by AlphaFold2 [35], and the high sequence conservation in mammals, 130 PRM*^Px+P^*-containing targets were selected for gene ontology (GO) analysis [36] (Figure 4A and Table S2). Consistent with the physiological roles of ASAP and GRAF proteins [21,37,38,39,40], these potential SH3*^AGS^*-interacting targets have their functions concentrated on actin cytoskeleton organization and vesicle-mediated transport (Figure 4A). These PRM*^Px+P^*-containing proteins also participate in other cellular membrane/cytoskeleton related functions, such as endocytosis, organelle localization, integrin-mediated signaling pathway, regulation of synapse organization and regulation of small GTPase-mediated signal transduction (Figure 4A), further indicating that the novel high-affinity SH3*^AGS^* and PRM*^Px+P^* interactions play certain roles in these two cellular activities. 

Although it has not been reported to participate in actin cytoskeleton organization or vesicle trafficking, SKAP1 containing an N-terminal PH domain is a typical module to attach with cellular membrane structure, and suggests the potential involvement of SKAP1 in membrane-related functions [41]. Interestingly, the cellular cytoskeleton organization is critical for the vesicle trafficking. The functional convergence of the SH3*^AGS^*-containing proteins and PRM^Px+P^-containing proteins suggests that the SH3^AGS^/PRM^Px+P^ interaction provides the strong and specific linkage for these two classes of proteins to form the complexes in the regulation of intracellular vesicle transport. In addition to the GO analysis, the PPI analysis indicates that the PRM^Px+P^-containing targets can assemble into a tight protein–protein interaction network with the SH3*^AGS^*-contianing proteins (Figure 4B), further supporting that they may function together in cells.

## 3. Discussion

In summary, we identified the novel ASAP1-SH3/MICAL1-PRM interaction with the binding affinity much stronger than the typical SH3/PRM interaction. Our structural study revealed the molecular determinants of such a high-affinity recognition and defined their unique binding motifs. After the motif searching, a novel class of the high-affinity SH3*^AGS^*/PRM*^Px+P^* recognitions were identified. The functional GO analysis suggested these recognitions play roles in the cellular functions of actin cytoskeleton organization and vesicle trafficking (Figure 4C).

What is the functional benefit for the SH3*^AGS^*-containg proteins to have high-affinity partners with the PRM*^Px+P^* motif? One possibility is to provide binding specificity. Compared to the large number of the PRM*^PxφP^*-containing proteins [3,6], the number of the PRM*^Px+P^*-containing proteins is very limited. Hence, the high-affinity binding ensures the specific recognition of SH3*^AGS^* to the PRM*^Px+P^*-containing targets out of all PRM*^PxxP^*-containing proteins. We noted with interest that ASAP1 also contains several class-II PRMs in the proline-rich region prior to the SH3 domain (Figure S6), which may bind to ASAP1-SH3 with low affinity like the typical PxφP-cored class-II PRM (Figure 2J and Figure S2). It suggests that ASAP1-SH3 may intrinsically bind to its own proline-rich region, which can be competitively destroyed by the MICAL1-PRM to form the stable complex of ASAP1 and MICAL1, as well as release the proline-rich region for recognition by other SH3-containing proteins.

Interestingly, several PRMs were recently identified to bind with the SH3 domains with relatively high affinity of *K*_d_ ~0.5–10 μM. However, they contain either a non-canonical sequence instead of the canonical PxxP motif (e.g., IRTKS-SH3/EspF-PRM) [42], or a special franking sequence outside the cored PxxP motif (e.g., PEP-3BP1/Csk-SH3) [43]). Unlike these cases, the PRM*^Px+P^* utilizes an unusual positively charged lysine or arginine at position 2 of the PxxP core to provide the additional interaction with the SH3*^AGS^* to enhance the binding affinity. Correspondingly, to accommodate the core sequence of the PRM*^Px+P^*, the SH3*^AGS^* domain employs a unique Cxx(D/E) sequence in the RT-loop to alter the central cavity for PRM binding with more specificity. 

The SH3*^AGS^*/PRM*^Px+P^* recognitions reported here show the adaptability and divergent strategies of target recognition by SH3 domains to achieve diverse functions. In addition to the protein ligand recognition, alternative modes of lipid recognition have been reported for SH3 domains [44,45]. Although SH3 domains have been extensively studied for decades, the answer to the specific SH3/ligand interactions remains incomplete.

## 4. Materials and Methods

### 4.1. Plasmids

The full-length mouse ASAP1, human MICAL1, human Spire1 and the fragmented human GRAF2-SH3 (residue 728–786), SKAP1-SH3 (residue 294–355), FAK1-PRM (residue 710–724) and FYB1-PRM (residue 362–376) genes were amplified by PCR from cDNAs. For protein overexpression in *E.coli*, the proteins were recombined into the modified pET32a vector with an N-terminal tandem Trx-His6 tag followed by a 3C protease cleavage cite. For cell transfection, MICAL1, ASAP1, Spire1 and MyoVa-GTD (residue 1318–1877) were recombined into a modified pcDNA3.1 with an N-terminal Flag tag or pEGFP-C1 vectors, respectively, as indicated. All mutants were performed by one-step PCR according to the QuikChange site-directed mutagenesis strategy (Vazyme Mut Express II Fast Mutagenesis Kit). All the plasmids were verified by DNA sequencing.

### 4.2. Cell Cultures and Transfection

Human 293T cells were maintained and cultured in Dulbecco’s Modified Eagle’s Medium (DMEM) supplemented with 10% Fetal Bovine Serum (FBS) and 1% Penicillin-Streptomycin Solution at 37 °C with 5% CO_2_. Lipofectamine 3000 reagent (Thermo Fisher Scientific, Waltham, MA, USA) was used for cell transfection following the instruction.

### 4.3. Co-Immunoprecipitation (Co-IP) Experiments

293T cells cotransfected with the indicated plasmids were harvested after 24-h transfection. The cells were lysed in a lysis buffer containing 50 mM Tris, pH 7.5, 100 mM NaCl, 1% Triton X-100, and protease inhibitors. The supernatant was obtained by centrifugation for 30 min at the speed of 15,000 rpm, and were then incubated with GFP beads for 1 h at 4 °C followed by three-time washing with the lysis buffer. Finally, the GFP beads with the bound proteins were mixed with loading buffer and boiled at 100 °C for 10 min, and detected by Western blotting. 

### 4.4. Protein Expression and Purification

For protein expression, the BL21(DE3) *E.coli* cells transfected with the plasmid of target protein were grown to OD600 of 0.6 in LB medium at 37 °C and then induced with 1 mM final concentration of isopropyl-1-thio-β-D-galactopyranoside (IPTG). After additional 16-h induction at 16 °C, the cells were collected by centrifugation at 5000 rpm for 15 min at 4 °C. For protein purification, the pellets were suspended in the lysis buffer containing 50 mM Tris, pH 7.5, 500 mM NaCl, 5 mM imidazole and then lysed using high pressure homogenizer. With centrifugation at 20,000 rpm for 30 min at 4 °C to remove the precipitates, the supernatant was loaded to the Ni-NTA column pre-equilibrated with the lysis buffer. After 30-min incubation, the column was washed three times by wash buffer containing 50 mM Tris, pH 7.5, 500 mM NaCl, 5 mM imidazole, and the target protein was eluted in 50 mM Tris, pH 7.5, 500 mM NaCl, 500 mM imidazole, which was further purified by Superdex-200 gel filtration on an ÄKTA Prime system (GE Healthcare) in the buffer containing 50 mM Tris, pH 7.5, 100 mM NaCl, 1 mM DTT, 1 mM EDTA. Finally, the eluted fractions were analyzed by SDS-PAGE and the target protein was concentrated for next experiments. 

### 4.5. Isothermal Titration Calorimetry (ITC)

ITC measurements were performed using a PEAQ-ITC Microcal calorimeter (Malvern, Northampton). The proteins were dissolved in the same buffer (50 mM Tris, pH 7.5, 100 mM NaCl, 1 mM DTT, 1 mM EDTA) and prepared with a concentration of 400 μM in the syringe and 40 μM in the cell. The titration was processed by injecting 3 μL of the sample in the syringe to the cell each time. An interval of 150 s between two injections was set to ensure the curve back to the baseline. The titration data were processed by MicroCal PEAQ-ITC Analysis Software and fitted by a one-site binding model. 

### 4.6. Analytical Size Exclusion Chromatography (aSEC) 

aSEC was carried out on an ÄKTA pure system (GE Healthcare). Protein sample was prepared with a final concentration of 50 μM and loaded onto a Superdex 200 Increase 10/300 GL column (GE Healthcare), equilibrated with a buffer containing 50 mM Tris, pH 7.5, 100 mM NaCl, 1 mM EDTA, and 1 mM DTT.

### 4.7. Crystallization and X-ray Data Collection

The purified MICAL1-PRM (residue 828–842) and ASAP1-SH3 (residue 1087–1147) were mixed at a ratio of 1.2:1 and incubated on ice for 30 min. The complex was concentrated to 15 mg/mL for crystal screening by using several commercial sparse matrix screens (Hampton research, Aliso Viejo, CA, USA) with the sitting drop vapor diffusion method at 16 °C. Finally, the crystals were obtained in the condition containing 0.1 M HEPES, pH 7.5, 1.4 M Sodium citrate tribasic dihydrate. After flash-cooling in liquid nitrogen with a crystallization solution containing 30% glycerol as cryo-protection, the crystals were delivered to collect the diffraction data at Shanghai Synchrotron Radiation Facility beamline BL19U1, and processed and scaled by the HKL3000 software package [46].

### 4.8. Structure Determination and Analysis

The initial phase was determined by using the molecular replacement method with the apo ASAP1-SH3 [PDB ID: 2RQT] as a search model, and then MICAL1-PRM was manually built to obtain the initial complex model. The complex structure was manually improved in COOT [47] and automatically refined by PHENIX refinement [48]. In the final stage, an additional TLS (translation, libration and screw-rotation) refinement was performed in PHENIX. The model quality was check by MolProbity [49]. The refinement statistics are listed in Table S1. All structure figures were prepared using PyMOL 2.0 (http://www.pymol.org/ accessed on 1 December 2022).

### 4.9. Motif Searching

The SH3 domains containing a unique Cxx(D/E) sequence were manually found by using Simple Modular Architecture Research Tool 4.0 (http://smart.embl.de accessed on 1 December 2022) [29]. The Scansite 4.0 web server was used to identify the PRM*^Px+P^*-containing proteins with searching the sequence pattern of xPx(K/R)Px(K/R) (https://scansite4.mit.edu/ accessed on 1 December 2022) [34], where x indicates any amino acid. 

### 4.10. Gene Ontology (GO) Analysis and Protein-Protein Interaction (PPI) Network Establishment 

Metascape [50] (https://metascape.org/ accessed on 1 December 2022) was used to perform GO biology process enrichment analysis. Terms with a *p*-value < 0.01, a minimum count of 3, and an enrichment factor >1.5 are collected and grouped into the clusters based on their membership similarities. The Search Tool for the Retrieval of Interacting Genes/Proteins (STRING) database [51] was used to analyze the PPI network of the above genes, and Cytoscape [52] was used to identify the network modules.

## Data Availability

The map and the atomic model of ASAP1-SH3 and MICAL1-PRM have been also deposited in PDB with accession code 8HLO.

## Supplementary Materials

The following supporting information can be downloaded at: https://www.mdpi.com/article/10.3390/ijms24021414/s1.

## References

[1] Slater O., Miller B., Kontoyianni M. (2020). Decoding Protein-protein Interactions: An Overview. Curr. Top. Med. Chem..

[2] Reimand J., Hui S., Jain S., Law B., Bader G.D. (2012). Domain-mediated protein interaction prediction: From genome to network. FEBS Lett..

[3] Kaneko T., Li L., Li S.S. (2008). The SH3 domain—A family of versatile peptide- and protein-recognition module. Front. Biosci..

[4] Mayer B.J. (2001). SH3 domains: Complexity in moderation. J. Cell Sci..

[5] Dionne U., Percival L.J., Chartier F.J.M., Landry C.R., Bisson N. (2022). SRC homology 3 domains: Multifaceted binding modules. Trends Biochem. Sci..

[6] Saksela K., Permi P. (2012). SH3 domain ligand binding: What’s the consensus and where’s the specificity?. FEBS Lett..

[7] Feng S., Chen J.K., Yu H., Simon J.A., Schreiber S.L. (1994). Two binding orientations for peptides to the Src SH3 domain: Development of a general model for SH3-ligand interactions. Science.

[8] Lim W.A., Richards F.M., Fox R.O. (1994). Structural determinants of peptide-binding orientation and of sequence specificity in SH3 domains. Nature.

[9] Goudreau N., Cornille F., Duchesne M., Parker F., Tocque B., Garbay C., Roques B.P. (1994). NMR structure of the N-terminal SH3 domain of GRB2 and its complex with a proline-rich peptide from Sos. Nat. Struct. Biol..

[10] Arold S., Franken P., Strub M.P., Hoh F., Benichou S., Benarous R., Dumas C. (1997). The crystal structure of HIV-1 Nef protein bound to the Fyn kinase SH3 domain suggests a role for this complex in altered T cell receptor signaling. Structure.

[11] Tame J.R., Sleigh S.H., Wilkinson A.J., Ladbury J.E. (1996). The role of water in sequence-independent ligand binding by an oligopeptide transporter protein. Nat. Struct. Biol..

[12] Lee C.H., Leung B., Lemmon M.A., Zheng J., Cowburn D., Kuriyan J., Saksela K. (1995). A single amino acid in the SH3 domain of Hck determines its high affinity and specificity in binding to HIV-1 Nef protein. Embo J..

[13] Deng L., Velikovsky C.A., Swaminathan C.P., Cho S., Mariuzza R.A. (2005). Structural basis for recognition of the T cell adaptor protein SLP-76 by the SH3 domain of phospholipase Cgamma1. J. Mol. Biol..

[14] Viguera A.R., Arrondo J.L., Musacchio A., Saraste M., Serrano L. (1994). Characterization of the interaction of natural proline-rich peptides with five different SH3 domains. Biochemistry.

[15] Yu H., Chen J.K., Feng S., Dalgarno D.C., Brauer A.W., Schreiber S.L. (1994). Structural basis for the binding of proline-rich peptides to SH3 domains. Cell.

[16] Knudsen B.S., Zheng J., Feller S.M., Mayer J.P., Burrell S.K., Cowburn D., Hanafusa H. (1995). Affinity and specificity requirements for the first Src homology 3 domain of the Crk proteins. EMBO J..

[17] Rubini C., Ruzza P., Spaller M.R., Siligardi G., Hussain R., Udugamasooriya D.G., Bellanda M., Mammi S., Borgogno A., Calderan A. (2010). Recognition of lysine-rich peptide ligands by murine cortactin SH3 domain: CD, ITC, and NMR studies. Biopolymers.

[18] Wu X., Knudsen B., Feller S.M., Zheng J., Sali A., Cowburn D., Hanafusa H., Kuriyan J. (1995). Structural basis for the specific interaction of lysine-containing proline-rich peptides with the N-terminal SH3 domain of c-Crk. Structure.

[19] Rouka E., Simister P.C., Janning M., Kumbrink J., Konstantinou T., Muniz J.R., Joshi D., O’Reilly N., Volkmer R., Ritter B. (2015). Differential Recognition Preferences of the Three Src Homology 3 (SH3) Domains from the Adaptor CD2-associated Protein (CD2AP) and Direct Association with Ras and Rab Interactor 3 (RIN3). J. Biol. Chem..

[20] Niu F., Sun K., Wei W., Yu C., Wei Z. (2020). F-actin disassembly factor MICAL1 binding to Myosin Va mediates cargo unloading during cytokinesis. Sci. Adv..

[21] Tanna C.E., Goss L.B., Ludwig C.G., Chen P.W. (2019). Arf GAPs as Regulators of the Actin Cytoskeleton-An Update. Int. J. Mol. Sci..

[22] Wang J., Morita Y., Mazelova J., Deretic D. (2012). The Arf GAP ASAP1 provides a platform to regulate Arf4- and Rab11-Rab8-mediated ciliary receptor targeting. EMBO J..

[23] Chen P.W., Billington N., Maron B.Y., Sload J.A., Chinthalapudi K., Heissler S.M. (2020). The BAR domain of the Arf GTPase-activating protein ASAP1 directly binds actin filaments. J. Biol. Chem..

[24] Hammer J.A., Sellers J.R. (2011). Walking to work: Roles for class V myosins as cargo transporters. Nat. Rev. Mol. Cell Biol..

[25] Pylypenko O., Welz T., Tittel J., Kollmar M., Chardon F., Malherbe G., Weiss S., Michel C.I., Samol-Wolf A., Grasskamp A.T. (2016). Coordinated recruitment of Spir actin nucleators and myosin V motors to Rab11 vesicle membranes. eLife.

[26] Brown M.T., Andrade J., Radhakrishna H., Donaldson J.G., Cooper J.A., Randazzo P.A. (1998). ASAP1, a phospholipid-dependent arf GTPase-activating protein that associates with and is phosphorylated by Src. Mol. Cell. Biol..

[27] Suzuki T., Nakamoto T., Ogawa S., Seo S., Matsumura T., Tachibana K., Morimoto C., Hirai H. (2002). MICAL, a novel CasL interacting molecule, associates with vimentin. J. Biol. Chem..

[28] Matsui C., Kaieda S., Ikegami T., Mimori-Kiyosue Y. (2008). Identification of a link between the SAMP repeats of adenomatous polyposis coli tumor suppressor and the Src homology 3 domain of DDEF. J. Biol. Chem..

[29] Letunic I., Khedkar S., Bork P. (2021). SMART: Recent updates, new developments and status in 2020. Nucleic Acids Res..

[30] Liu Y., Loijens J.C., Martin K.H., Karginov A.V., Parsons J.T. (2002). The association of ASAP1, an ADP ribosylation factor-GTPase activating protein, with focal adhesion kinase contributes to the process of focal adhesion assembly. Mol. Biol. Cell.

[31] Hildebrand J.D., Taylor J.M., Parsons J.T. (1996). An SH3 domain-containing GTPase-activating protein for Rho and Cdc42 associates with focal adhesion kinase. Mol. Cell. Biol..

[32] Liu J., Kang H., Raab M., da Silva A.J., Kraeft S.K., Rudd C.E. (1998). FYB (FYN binding protein) serves as a binding partner for lymphoid protein and FYN kinase substrate SKAP55 and a SKAP55-related protein in T cells. Proc. Natl. Acad. Sci. USA.

[33] Lucken-Ardjomande Hasler S., Vallis Y., Pasche M., McMahon H.T. (2020). GRAF2, WDR44, and MICAL1 mediate Rab8/10/11-dependent export of E-cadherin, MMP14, and CFTR DeltaF508. J. Cell Biol..

[34] Obenauer J.C., Cantley L.C., Yaffe M.B. (2003). Scansite 2.0: Proteome-wide prediction of cell signaling interactions using short sequence motifs. Nucleic Acids Res..

[35] Jumper J., Evans R., Pritzel A., Green T., Figurnov M., Ronneberger O., Tunyasuvunakool K., Bates R., Zidek A., Potapenko A. (2021). Highly accurate protein structure prediction with AlphaFold. Nature.

[36] Ashburner M., Ball C.A., Blake J.A., Botstein D., Butler H., Cherry J.M., Davis A.P., Dolinski K., Dwight S.S., Eppig J.T. (2000). Gene ontology: Tool for the unification of biology. The Gene Ontology Consortium. Nat. Genet..

[37] Sabe H., Onodera Y., Mazaki Y., Hashimoto S. (2006). ArfGAP family proteins in cell adhesion, migration and tumor invasion. Curr. Opin. Cell Biol..

[38] Vitali T., Girald-Berlingeri S., Randazzo P.A., Chen P.W. (2019). Arf GAPs: A family of proteins with disparate functions that converge on a common structure, the integrin adhesion complex. Small GTPases.

[39] Lucken-Ardjomande Hasler S., Vallis Y., Jolin H.E., McKenzie A.N., McMahon H.T. (2014). GRAF1a is a brain-specific protein that promotes lipid droplet clustering and growth, and is enriched at lipid droplet junctions. J. Cell Sci..

[40] Shibata H., Oishi K., Yamagiwa A., Matsumoto M., Mukai H., Ono Y. (2001). PKNbeta interacts with the SH3 domains of Graf and a novel Graf related protein, Graf2, which are GTPase activating proteins for Rho family. J. Biochem..

[41] Raab M., Smith X., Matthess Y., Strebhardt K., Rudd C.E. (2011). SKAP1 protein PH domain determines RapL membrane localization and Rap1 protein complex formation for T cell receptor (TCR) activation of LFA-1. J. Biol. Chem..

[42] Aitio O., Hellman M., Kazlauskas A., Vingadassalom D.F., Leong J.M., Saksela K., Permi P. (2010). Recognition of tandem PxxP motifs as a unique Src homology 3-binding mode triggers pathogen-driven actin assembly. Proc. Natl. Acad. Sci. USA.

[43] Ghose R., Shekhtman A., Goger M.J., Ji H., Cowburn D. (2001). A novel, specific interaction involving the Csk SH3 domain and its natural ligand. Nat. Struct. Biol..

[44] Perez Y., Maffei M., Igea A., Amata I., Gairi M., Nebreda A.R., Bernado P., Pons M. (2013). Lipid binding by the Unique and SH3 domains of c-Src suggests a new regulatory mechanism. Sci. Rep..

[45] Toke O., Koprivanacz K., Radnai L., Mero B., Juhasz T., Liliom K., Buday L. (2021). Solution NMR Structure of the SH3 Domain of Human Caskin1 Validates the Lack of a Typical Peptide Binding Groove and Supports a Role in Lipid Mediator Binding. Cells.

[46] Minor W., Cymborowski M., Otwinowski Z., Chruszcz M. (2006). HKL-3000: The integration of data reduction and structure solution—From diffraction images to an initial model in minutes. Acta Crystallogr. D Biol. Crystallogr..

[47] Emsley P., Lohkamp B., Scott W.G., Cowtan K. (2010). Features and development of Coot. Acta Crystallogr. D Biol. Crystallogr..

[48] Adams P.D., Afonine P.V., Bunkoczi G., Chen V.B., Davis I.W., Echols N., Headd J.J., Hung L.W., Kapral G.J., Grosse-Kunstleve R.W. (2010). PHENIX: A comprehensive Python-based system for macromolecular structure solution. Acta Crystallogr. D Biol. Crystallogr..

[49] Chen V.B., Arendall W.B., Headd J.J., Keedy D.A., Immormino R.M., Kapral G.J., Murray L.W., Richardson J.S., Richardson D.C. (2010). MolProbity: All-atom structure validation for macromolecular crystallography. Acta Crystallogr. D Biol. Crystallogr..

[50] Zhou Y., Zhou B., Pache L., Chang M., Khodabakhshi A.H., Tanaseichuk O., Benner C., Chanda S.K. (2019). Metascape provides a biologist-oriented resource for the analysis of systems-level datasets. Nat. Commun..

[51] Szklarczyk D., Gable A.L., Nastou K.C., Lyon D., Kirsch R., Pyysalo S., Doncheva N.T., Legeay M., Fang T., Bork P. (2021). The STRING database in 2021: Customizable protein-protein networks, and functional characterization of user-uploaded gene/measurement sets. Nucleic Acids Res..

[52] Shannon P., Markiel A., Ozier O., Baliga N.S., Wang J.T., Ramage D., Amin N., Schwikowski B., Ideker T. (2003). Cytoscape: A software environment for integrated models of biomolecular interaction networks. Genome Res..

