# Supplementary material for: Crystal Structure of the SH3 Domain of ASAP1 in Complex with the Proline Rich Motif (PRM) of MICAL1 Reveals a Unique SH3/PRM Interaction Mode
Source: Int J Mol Sci. 2023 Jan 11;24(2):1414. doi: 10.3390/ijms24021414 (PMC9865144; doi:10.3390/ijms24021414)
Supplement: Supplementary file 1 [file ijms-24-01414-s001.zip › ijms-2105266-supplementary.pdf]

International Journal of
Molecular Sciences

MDPI

Supplementary Figures and Tables:

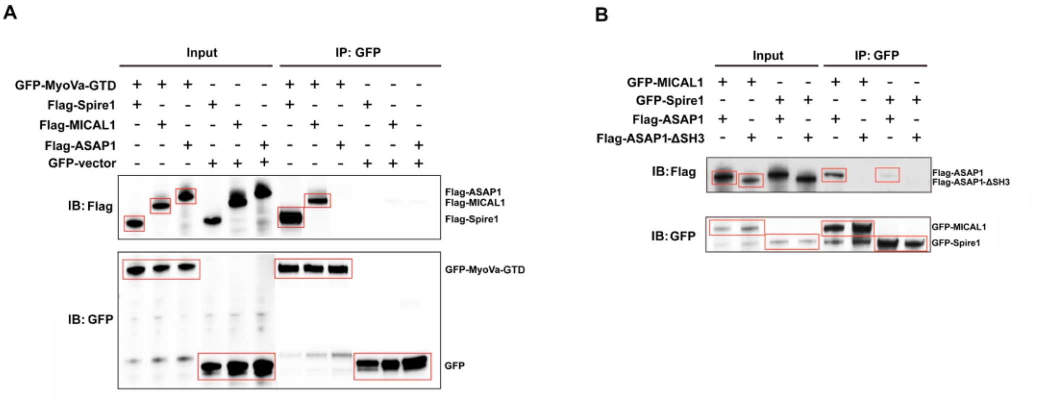

Figure S1. MICAL1 interacts with ASAP1. (**A**) Co-IP result showing that ASAP1 cannot associate with MyoVa-GTD in 293T cells, differing from MICAL1 and Spire1. (**B**) Co-IP result showing that MICAL1, stronger than Spire1, interacts with ASAP1.

SH3 domain-containing protein	PRM -containing protein	Binding affinity (μM)	PRM Sequence PxφPx(K/R)	Measurement Method	Reference
Grab2	C3G	142	PPPALPPKKR	Fluorescence spectroscopy	Ref-18
v-Crk	Sos	23.7	PPPVPPRRRR	Fluorescence spectroscopy	Ref-18
Src	PLR1	59	AFAPPLPRR	Fluorescence spectroscopy	Ref-7
Hck	Nef	91	VRPQVPLRPM	Surface plasmon resonance	Ref-12
SEM-5	Sos	43	PPPVPPRRRR	Fluorescence spectroscopy	Ref-8
Fyn	Nef	202	VRPQVPLRPM	Fluorescence spectroscopy	Ref-12
Cortactin	HPK1	64	KPPLLPPKKE	Isothermal titration calorimetry	Ref-17
PLCγ1	SLP-76	33	TPPVPPQRPM	Isothermal titration calorimetry	Ref-13

Figure S2. List of some typical class-II PRMs targeted by the SH3-containing proteins with relatively low binding affinities. The reported sequence of the class-II PRMs and their binding affinities to the SH3 domains as well as the affinity detection method are summarized.

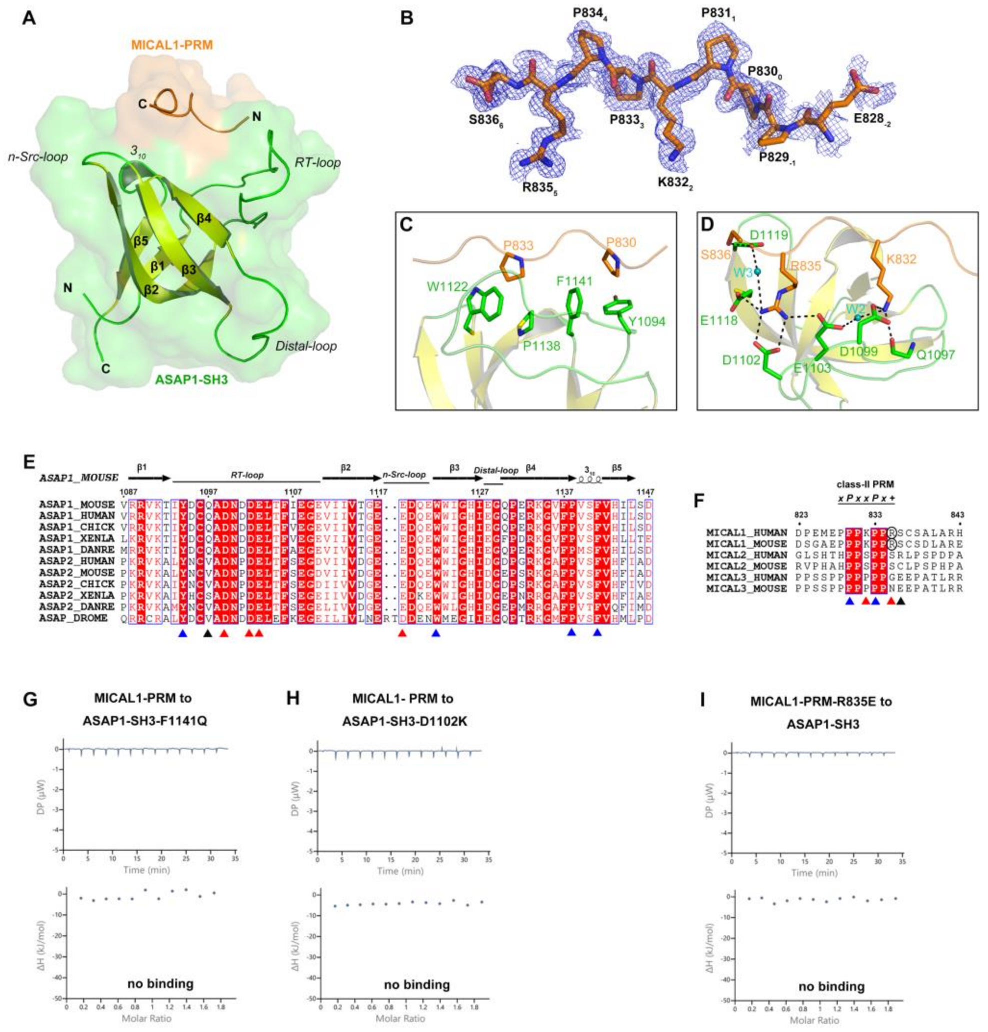

Figure S3. Overall structure of ASAP1-SH3 and MICAL1-PRM complex. (**A**) Crystal structure of ASAP1-SH3 and MICAL1-PRM complex in cartoon mode covered with a transparent surface. The conserved five β-sheets, three loops and a short helix in ASAP1-SH3 are labeled. (**B**) The F_o-F_c map of MICAL1-PRM with its atomic model in stick mode. (**C** and **D**) The detailed contacts of ASAP1-SH3 and MICAL1-PRM, including the hydrophobic (**C**) and charge-to-charge (**D**) interactions. (**E** and **F**) Sequence alignments of the SH3 domains in ASAP family proteins (**E**) and the proline-rich regions in MICAL family proteins (**F**). The residues involving hydrophobic, charge-to-charge and hydrogen bonding interactions observed in ASAP1-SH3/MICAL1-PRM structure are labeled by blue, red, and black triangles, respectively. The sequence pattern of class-II PRM is also indicated in **F**. (**G** to **I**) ITC-based affinity measurements of either mutated ASAP1-SH3 (**G**, **H**) or mutated MICAL1-PRM (**I**) binding to the wild-type proteins as indicated.

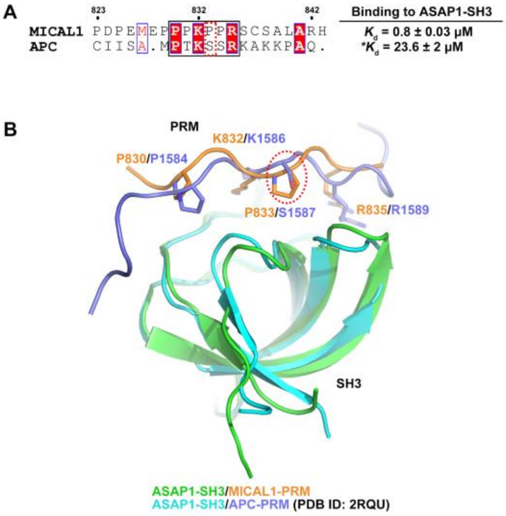

Figure S4. Sequence and structure comparisons of ASAP-SH3 binding to MICAL1-PRM or APC-PRM. (**A**) Sequence alignment showing the difference PRMs with different affinity binding to ASAP1-SH3. *The reported affinity in another studies. (**B**) Structural superimposing by aligning their SH3 domain structures. The different residues in MICAL1-PRM and APC-PRM are circled by a red dash line.

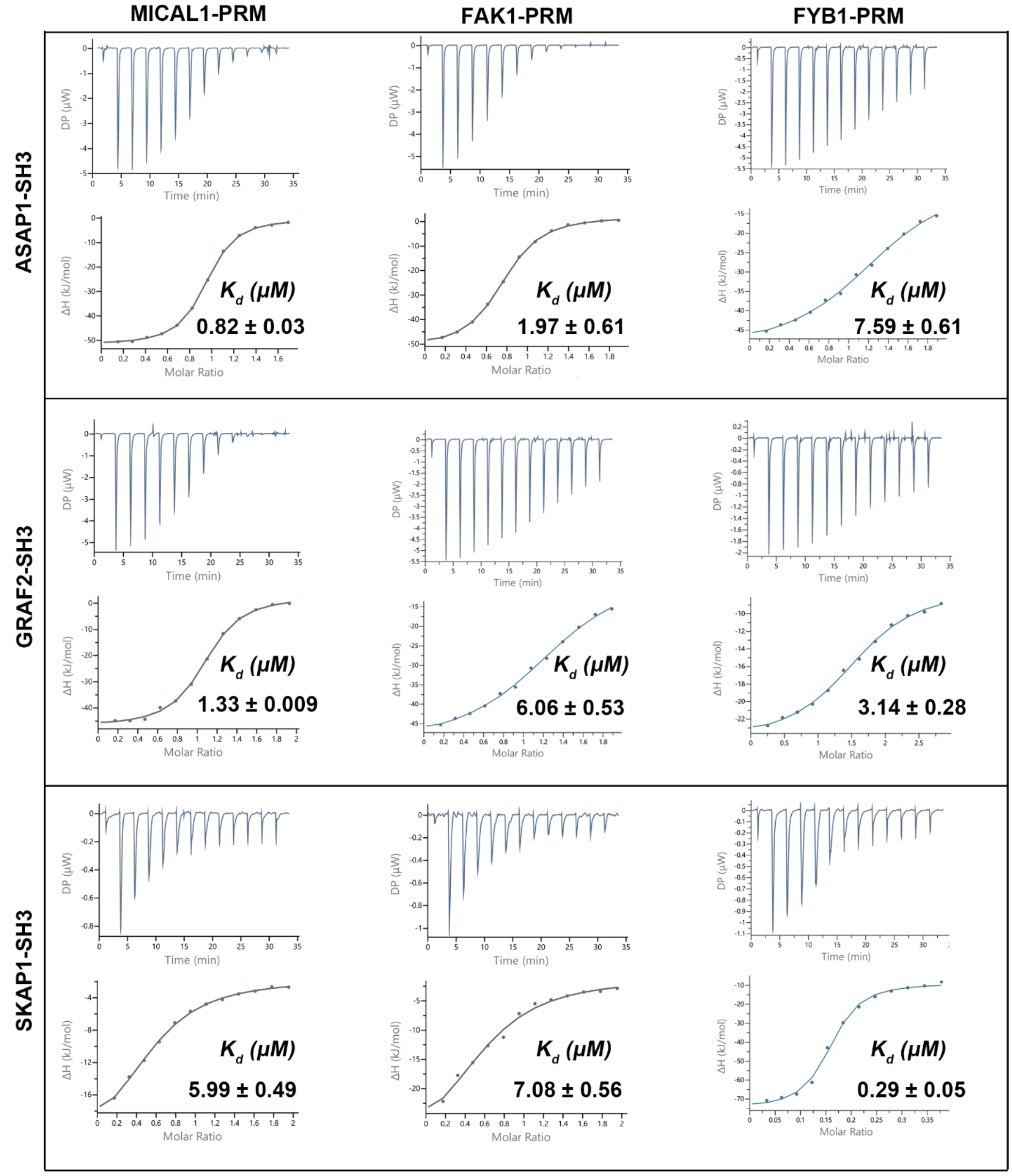

Figure S5. ITC curves showing the interactions between the SH3 domains of ASAP1/GRAF2/SKAP1 and the PRMs of MICAL1/FAK1/FYB1, summarized in Figure3C.

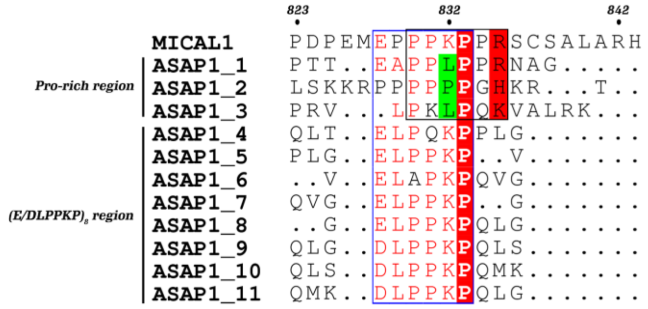

Figure S6. Sequence alignment of the proline-rich region and 8*(E/D)LPPKP region in ASAP1 with MICAL1-PRM. Three typical class-II PRMs can be found in proline-rich region, which contain a hydrophobic residue (green) in the position corresponding K832 of MICAL1-PRM. All eight (E/D)LPPKP motifs is lack of the positively charged residue in the position corresponding R835 in MICAL1-PRM.

Table S1. Data collection and refinement statistics.

**Data collection**	
X-ray source	SSRF-19U1
Space group	$P6_2$
Cell dimensions	
a, b, c (Å)	45.18, 45.18, 59.39
α, β, γ (°)	90.00, 90.00, 120.00
Resolution range (Å)	39.13 – 1.17 (1.21 – 1.17)
R_{merge} (%)[a]	4.1 (19.16)
$CC_{1/2}$[b]	0.99 (0.90)
$I/\sigma(I)$	11.32 (2.50)
Completeness (%)	91.7 (48.54)
Redundancy	4.9 (4.6)
**Refinement**	
Resolution (Å)	39.13 – 1.17 (1.21 – 1.17)
No. Reflections	21440 (1130)
R_{work}/R_{free} (%)[c]	11.3 (15.6) / 14.2 (20.7)
No. atoms	
Protein	657
Ligand	5
Water	114
Mean B (Å^2)	20.5
R.m.s deviations	
Bond lengths (Å)	0.008
Bond angles (º)	1.31
Ramachandran analysis	
Favored region (%)	98.7
Allowed region (%)	1.3
Outliers (%)	0

The numbers in parentheses represent values for the highest resolution shell.

[a]$R_{merge} = \sum|I_i - I_m|/\sum I_i$, where Ii is the intensity of the measured reflection and I_m is the mean intensity of all symmetry related reflections.

[b]$CC_{1/2}$ is the correlation coefficient of the half datasets.

[c]$R_{work} = \Sigma||F_{obs}| - |F_{calc}||/\Sigma|F_{obs}|$, where F_{obs} and F_{calc} are observed and calculated structure factors.

$R_{free} = \Sigma_T||F_{obs}| - |F_{calc}||/\Sigma_T|F_{obs}|$, where T is a test data set of about 1.7 % of the total reflections randomly chosen and set aside prior to refinement.

Table S2. List of the PRM^{Px+P}-containing proteins for GO analysis.

Gene Name	Protein Name	Uniprot ID	GO analysis-suggested term ID and functional field	PRM Position	PRM Sequence
ABCF1	ABCF1	Q8NE71		112-125	ALLLPMKPLKATAT
ANS1A	ANS1A	Q92625		834-847	YEEPPQKPPRFSQL
ANS1B	ANS1B	Q7Z6G8		948-961	HDDPPQKPPRSITL
ARAP3	ARAP3	Q8WWN8	GO:0022604 (regulation of cell morphogenesis); GO:1902532 (negative regulation of intracellular signal transduction)	388-401	RPPQPPRPLRTGML
ARHGEF5	ARHG5	Q12774	GO:0051493 (regulation of cytoskeleton organization)	628-641	HSELPQRPPKPAIY
ARHGEF12	ARHG C	Q9NZN5	GO:0045859 (regulation of protein kinase activity)	600-613	ILGPPRRPSRHDNS
ASPSCR1	ASPC1	Q9BZE9		275-388	SPGGPSKPKKSKSG
ATP2A2	AT2A2	P16615	GO:0060627 (regulation of vesicle-mediated transport); GO:0051640 (organelle localization)	496-509	VYCTPNKPSRTSMS
SLC4A3	B3A3	P48751		55-68	PAWDPEKPSRSYSE
BCAR3	BCAR3	O75815	GO:1901699 (cellular response to nitrogen compound)	400-413	CPKPPPKPCKVPFL
BMPR2	BMPR2	Q13873	GO:0045859 (regulation of protein kinase activity); GO:0120035 (regulation of plasma membrane bounded cell projection organization); GO:0008285 (negative regulation of cell population proliferation); GO:0071363 (cellular response to growth factor stimulus)	925-938	ADPGPSKPRRAQRP
CDC42EP2	BORG1	O14613	GO:0051493 (regulation of cytoskeleton organization); GO:0120035 (regulation of plasma membrane bounded cell projection organization);	123-136	TAQAPPKPPRLHLE

			GO:0022604 (regulation of cell morphogenesis); GO:0120034 (positive regulation of plasma membrane bounded cell projection assembly)		
BRPF1	BRPF1	P55201		901-914	TAGPPKRPGRPPKN
BTG2	BTG2	P78543	GO:0009896 (positive regulation of catabolic process); GO:0008285 (negative regulation of cell population proliferation); GO:0031175 (neuron projection development)	52-65	HHWFPEKPSKGSGY
BTG1	BTG1	P62324	GO:0008285 (negative regulation of cell population proliferation)	54-67	HHWFPEKPCKGSGY
RLTPR	CARL2	Q6F5E8	GO:0120035 (regulation of plasma membrane bounded cell projection organization); GO:0120034 (positive regulation of plasma membrane bounded cell projection assembly)	1135-1148	LLRPPTRPSRGEEL
LRRC16B	CARL3	Q8ND23		1334-1347	RRTAPLKPKRTRRA
PRKCDBP	CAVN3	Q969G5	GO:1902532 (negative regulation of intracellular signal transduction)	202-215	PPPTPVKPPRLGPG
CBLB	CBLB	Q13191	GO:0009896 (positive regulation of catabolic process); GO:0045859 (regulation of protein kinase activity); GO:0050852 (T cell receptor signaling pathway); GO:0008285 (negative regulation of cell population proliferation); GO:0042326 (negative regulation of phosphorylation)	898-911	GSQAPARPPKPRPR
CD2AP	CD2AP	Q9Y5K6	GO:0051493 (regulation of cytoskeleton organization); GO:0009896 (positive regulation of catabolic process); GO:1902532 (negative regulation of intracellular signal transduction)	406-419	GTVYPKRPEKPVPP

CDKN1B	CDN1B	P46527	GO:0051493 (regulation of cytoskeleton organization); GO:0045859 (regulation of protein kinase activity); GO:0008285 (negative regulation of cell population proliferation); GO:0042326 (negative regulation of phosphorylation)	87-100	FYYRPPRPPKGACK
CHD4	CHD4	Q14839	GO:0050808 (synapse organization)	1380-1393	RSEAPRRPSRKGLR
CHMP4B	CHM4B	Q9H444	GO:0051668 (localization within membrane); GO:0051668 (localization within membrane); GO:0051640 (organelle localization)	196-209	SIALPSKPAKKKEE
CLUAP1	CLUA1	Q96AJ1		389-402	ISLSPTKPNRRVRK
CASKIN1	CSKI1	Q8WXD9		869-882	ADAEPGRPKKRAHS
CASKIN2	CSKI2	Q8WXE0		787-800	TPPDPPRPKRRSHS
DENND1C	DEN1C	Q8IV53	GO:0051668 (localization within membrane)	504-517	GKNRPLRPSRRRQL
DENND4B	DEN4B	O75064	GO:0006897 (endocytosis)	510-523	PRTLPRRPYKVLLA
DLGAP1	DLGP1	O14490	GO:0098962 (regulation of postsynaptic neurotransmitter receptor activity); GO:1902532 (negative regulation of intracellular signal transduction)	913-926	PPPVPKKPAKGPAP
DLGAP2	DLGP2	Q9P1A6		988-1001	PPPIPKKPPKGKFP
DLGAP3	DLGP3	O95886	GO:0098962 (regulation of postsynaptic neurotransmitter receptor activity); GO:0050808 (synapse organization)	913-926	PPPIPKKPLRGRGV
DLGAP4	DLGP4	Q9Y2H0	GO:0098962 (regulation of postsynaptic neurotransmitter receptor activity)	926-939	PPPVPKKPAKSKPA
DLG5	DLG5	Q8TDM6	GO:0008285 (negative regulation of cell population proliferation)	1008-1021	GPLTPPKPPRRSDS

DOC2A	DOC2A	Q14183	GO:0060627 (regulation of vesicle-mediated transport); GO:0006897 (endocytosis)	21-34	VCPGPIRPIRQISD
DOC2B	DOC2B	Q14184		22-35	VCPGPIRPIKQISD
DOCK1	DOCK1	Q14185	GO:0022604 (regulation of cell morphogenesis)	1731-1744	ETISPLRPQRPKSQ
DOK7	DOK7	Q18PE1	GO:0045859 (regulation of protein kinase activity); GO:0050808 (synapse organization)	314-327	ASRPPPKPLRPRQL
DRD2	DRD2	P14416	GO:0045859 (regulation of protein kinase activity); GO:0006897 (endocytosis); GO:1901699 (cellular response to nitrogen compound); GO:0050808 (synapse organization); GO:0008285 (negative regulation of cell population proliferation); GO:1902532 (negative regulation of intracellular signal transduction); GO:0031175 (neuron projection development)	319-332	TPDSPAKPEKNGHA
DNAH1	DYH1	Q9P2D7		646-659	LINSPYRPRKNPLF
DNAH5	DYH5	Q8TE73		3927-3940	LKACPPKPSKWILD
DNM1	DYN1	Q05193	GO:0006897 (endocytosis); GO:1901699 (cellular response to nitrogen compound); GO:0051668 (localization within membrane); GO:0051640 (organelle localization)	829-842	PPQVPSRPNRAPPG
DNM2	DYN2	P50570	GO:0060627 (regulation of vesicle-mediated transport); GO:0120035 (regulation of plasma membrane bounded cell projection organization); GO:0006897 (endocytosis); GO:0022604 (regulation of cell morphogenesis); GO:0051640 (organelle localization);	828-841	FGAPPQVPSRPTRA

			GO:0120034 (positive regulation of plasma membrane bounded cell projection assembly);<br>GO:0031175 (neuron projection development)		
DNM23	DYN3	Q9UQ16	GO:0120035 (regulation of plasma membrane bounded cell projection organization);<br>GO:0006897 (endocytosis);<br>GO:0050808 (synapse organization);<br>GO:0120034 (positive regulation of plasma membrane bounded cell projection assembly)	831-844	PPQVPSRPTRAPPS
EGR1	EGR1	P18146	GO:0045859 (regulation of protein kinase activity);<br>GO:1901699 (cellular response to nitrogen compound);<br>GO:0071363 (cellular response to growth factor stimulus)	321-334	MRKYPNRPSKTPPH
EHD1	EHD1	Q9H4M9	GO:0060627 (regulation of vesicle-mediated transport); GO:0120035 (regulation of plasma membrane bounded cell projection organization);<br>GO:0006897 (endocytosis);<br>GO:0051668 (localization within membrane);<br>GO:0031175 (neuron projection development);<br>GO:0071363 (cellular response to growth factor stimulus)	114-127	LVVDPRRPFRKLNA
EHD2	EHD2	Q9NZN4	GO:0060627 (regulation of vesicle-mediated transport);<br>GO:0006897 (endocytosis);<br>GO:0051668 (localization within membrane)	114-127	LVVDPDKPFRKLNP
EHD3	EHD3	Q9NZN3	GO:0006897 (endocytosis);<br>GO:0051668 (localization within membrane)	114-127	LVVDPKKPFRKLNA
EHD4	EHD4	Q9H223	GO:0060627 (regulation of vesicle-mediated transport);	117-130	LVVDPKKPFRKLSR

			GO:0006897 (endocytosis); GO:0051668 (localization within membrane); GO:0071363 (cellular response to growth factor stimulus)		
ERRFI1	ERRFI	Q9UJM3	GO:0045859 (regulation of protein kinase activity); GO:1901699 (cellular response to nitrogen compound); GO:1902532 (negative regulation of intracellular signal transduction); GO:0042326 (negative regulation of phosphorylation); GO:0071363 (cellular response to growth factor stimulus)	286-299	RVPIPPRPVKPDYR
**PTK2**	**FAK1**	**Q05397**	**GO:0009896 (positive regulation of catabolic process); GO:0120035 (regulation of plasma membrane bounded cell projection organization); GO:1901699 (cellular response to nitrogen compound); GO:0022604 (regulation of cell morphogenesis); GO:0043551 (regulation of phosphatidylinositol 3-kinase activity); GO:0008285 (negative regulation of cell population proliferation); GO:0031175 (neuron projection development); GO:0071363 (cellular response to growth factor stimulus)**	**708-721**	**SDEAPPKPSRPGYP**
**PTK2B**	**FAK2**	**Q14289**	**GO:0051493 (regulation of cytoskeleton organization); GO:0009896 (positive regulation of catabolic process); GO:0045859 (regulation of protein kinase activity); GO:0043551 (regulation of phosphatidylinositol 3-kinase activity); GO:0031175 (neuron projection development)**	**710-723**	**FQEPPPKPSRPKYR**

FBXO7	FBX7	Q9Y3I1	GO:0009896 (positive regulation of catabolic process); GO:0045859 (regulation of protein kinase activity); GO:0120035 (regulation of plasma membrane bounded cell projection organization); GO:0042326：negative regulation of phosphorylation	502-515	NDRFPFRPSRGRPT
FGD5	FGD5	Q6ZNL6	GO:0022604 (regulation of cell morphogenesis)	787-800	IQIPPRRPARAGAF
FMN2	FMN2	Q9NZ56	GO:0051640 (organelle localization)	1285-1298	QPIEPCRPMKPLYW
FOXA2	FOXA2	Q9Y261	GO:0042326 (negative regulation of phosphorylation)	220-233	VPRSPDKPGKGSFW
**FYB**	**FYB1**	**O15117**	**GO:0051668 (localization within membrane); GO:0050852 (T cell receptor signaling pathway)**	**353-366**	**LGPPPPKPNRPPNV**
C1orf168	FYB2	Q5VWT5	GO:0051668 (localization within membrane); GO:0050852 (T cell receptor signaling pathway)	275-288	LGPPPPKPSRPPIV
FZR1	FZR1	Q9UM11	GO:0009896 (positive regulation of catabolic process)	160-173	LLRSPRKPTRKISK
GAL3ST3	G3ST3	Q96A11		413-426	LDNPPPRPIRVLPR
GAB1	GAB1	Q13480	GO:1901699 (cellular response to nitrogen compound); GO:0071363 (cellular response to growth factor stimulus)	338-351	PDIPPPRPPKPHPA
GAB2	GAB2	Q9UQC2	GO:0060627 (regulation of vesicle-mediated transport)	348-361	AIAPPPRPPKPSQA
GAB3	GAB3	Q8WWW8		304-317	SNTPPPRPPKPSHL
GABRA1	GBRA1	P14867	GO:0050808 (synapse organization)	347-360	KSVVPEKPKKVKDP
GPR158	GP158	Q5T848	GO:0051668 (localization within membrane)	963-976	PAEEPRKPQKSGIM
GPC2	GPC2	Q8N158	GO:0120035 (regulation of plasma membrane bounded cell projection organization)	516-529	AVAPPARPPRPPYP

HYPK	HYPK	Q9NX55		18-31	PERPPEKPRKHDSG
ISLR2	ISLR2	Q6UXK2	GO:0120035 (regulation of plasma membrane bounded cell projection organization)	617-630	LAKHPGKPYRLILR
ITCH	ITCH	Q96J02	GO:0009896 (positive regulation of catabolic process); GO:0008285 (negative regulation of cell population proliferation); GO:1902532 (negative regulation of intracellular signal transduction)	249-262	KPSRPPRPSRPPPP
KHDRBS1	KHDR1	Q07666	GO:0050852 (T cell receptor signaling pathway)	423-436	LKAPPARPVKGAYR
KIF26B	KI26B	Q2KJY2	GO:0051640 (organelle localization)	423-436	LKAPPARPVKGAYR
KIF1A	KIF1A	Q12756	GO:0120035 (regulation of plasma membrane bounded cell projection organization)	1853-1866	KITPPRRPHRCSSG
KIF23	KIF23	Q02241		1091-1104	DVLSPLRPSRRHFP
KNDC1	KNDC1	Q76NI1		824-837	GPRHPPKPPRSKAT
LARP1	LARP1	Q6PKG0	GO:0009896 (positive regulation of catabolic process); GO:1901699 (cellular response to nitrogen compound)	564-577	PRPSPARPKKSEES
LRFN1	LRFN1	Q9P244		519-532	GDPAPCRPLRAHFL
LRFN4	LRFN4	Q6PJG9	GO:0050808 (synapse organization)	573-586	PRSPPPRPQRSCSL
LRP1	LRP1	Q07954	GO:0051493 (regulation of cytoskeleton organization); GO:0009896 (positive regulation of catabolic process); GO:0060627 (regulation of vesicle-mediated transport); GO:0006897 (endocytosis); GO:1901699 (cellular response to nitrogen compound); GO:0042326 (negative regulation of phosphorylation)	3693-3706	FVCPPNRPFRCKND
LRRC7	LRRC7	Q96NW7		748-761	PPGNIPQRPDRLPMS

LSM14A	LS14A	Q8ND56		395-408	TFGIPLRPNRGRGGY
MAP1A	MAP1A	P78559	GO:0051493 (regulation of cytoskeleton organization)	367-380	SSEKPPEKPAKPERV
MAP6	MAP6	Q96JE9	GO:0051493 (regulation of cytoskeleton organization); GO:0120035 (regulation of plasma membrane bounded cell projection organization); GO:0051640 (organelle localization); GO:0031175 (neuron projection development)	309-322	TDIKPVKPIKAKPQ
**MICAL1**	**MICA1**	**Q8TDZ2**	**GO:0060627 (regulation of vesicle-mediated transport); GO:0042326 (negative regulation of phosphorylation)**	**826-839**	**EMEPPPKPPRSCSA**
MKRN2	MKRN2	Q9H000	GO:0009896 (positive regulation of catabolic process); GO:1902532 (negative regulation of intracellular signal transduction)	353-366	RLAEPEKPRKQLSS
SBF1	MTMR5	O95248		1031-1044	SAHTPGRPPRVTKD
MYO16	MYO16	Q9Y6X6	GO:0008285 (negative regulation of cell population proliferation); GO:0031175 (neuron projection development)	1293-1306	RKQPPPKPKRDPNT
MYO1C	MYO1C	O00159	GO:0051668 (localization within membrane); GO:0071363 (cellular response to growth factor stimulus)	15-28	RVVHPHRPCKLALG
MYO3A	MYO3A	Q8NEV4	GO:0051493 (regulation of cytoskeleton organization); GO:0120035 (regulation of plasma membrane bounded cell projection organization); GO:0120034 (positive regulation of plasma membrane bounded cell projection assembly)	1489-1502	ILRPPRRPRKPKTL
MYO5A	MYO5A	Q9Y4I1	GO:1901699 (cellular response to nitrogen compound);	612-625	LTRTPAKPTKGRPG

			GO:0051668 (localization within membrane); GO:0051640 (organelle localization)		
NDST2	NDST2	P52849		67-80	RPPVPPRPPRPPET
OSTF1	OSTF1	Q92882		1-14	MSKPPPKPVKPGQV
OTOF	OTOF	Q9HC10		1722-1735	LDISPRKPKKYELR
PIK3R3	P55G	Q92569	GO:0051493 (regulation of cytoskeleton organization); GO:0120035 (regulation of plasma membrane bounded cell projection organization); GO:1901699 (cellular response to nitrogen compound); GO:0043551 (regulation of phosphatidylinositol 3-kinase activity)	34-47	PPALPPKPPKPMTS
PIK3R1	P85A	P27986	GO:1901699 (cellular response to nitrogen compound); GO:0043551 (regulation of phosphatidylinositol 3-kinase activity); GO:0120034 (positive regulation of plasma membrane bounded cell projection assembly)	304-319	APALPPKPPKPTTV
PCDH12	PCD12	Q9NPG4		759-772	YRQQPKRPQKHIQK
PDGFRA	PGFRA	P16234	GO:0051493 (regulation of cytoskeleton organization); GO:1901699 (cellular response to nitrogen compound); GO:0043551 (regulation of phosphatidylinositol 3-kinase activity); GO:0071363 (cellular response to growth factor stimulus)	694-707	LSHHPEKPKKELDI
**PRAM1**	**PRAM**	**Q96QH2**	**GO:0060627 (regulation of vesicle-mediated transport); GO:0051668 (localization within membrane); GO:0050852 (T cell receptor signaling pathway)**	**112-125**	**VTDLPKKPSKLELSD**
PJA1	PJA1	Q8NG27		226-239	EQNLPARPSRAPVS

PSRC1	PSRC1	Q6PGN9	GO:0051493 (regulation of cytoskeleton organization); GO:0045859 (regulation of protein kinase activity); GO:0051640 (organelle localization)	34-47	VLVTPEKPLRRGLS
PTCH1	PTC1	Q13635	GO:0051668 (localization within membrane); GO:0008285 (negative regulation of cell population proliferation); GO:0031175 (neuron projection development)	1311-1324	LWPPPYRPRRDAFE
PTPN1	PTN1	P18031	GO:0009896 (positive regulation of catabolic process); GO:0045859 (regulation of protein kinase activity); GO:0060627 (regulation of vesicle-mediated transport); GO:1901699 (cellular response to nitrogen compound); GO:1902532 (negative regulation of intracellular signal transduction); GO:0042326 (negative regulation of phosphorylation); GO:0071363 (cellular response to growth factor stimulus)	305-318	HIPPPPRPPKRILE
PTPN12	PTN12	Q05209		330-353	KQDSPPPKPPRTRS
CAD	PYR1	P27708		1830-1843	MTTTPERPRRGIPG
REPS1	REPS1	Q96D71	GO:0006897 (endocytosis)	695-708	PLAPPPKPVRRRLK
RGS5	RGS5	O15539	GO:1901699 (cellular response to nitrogen compound)	40-53	YNEKPEKPAKTQKT
RGS8	RGS8	P57771	GO:1901699 (cellular response to nitrogen compound)	31-44	TAILPDKPNRALKR
ARHGAP32	RHG32	A7KAX9		1976-1989	SIVPPPKPERSHSL
BZRAP1	RIMB1	O95153	GO:0022604 (regulation of cell morphogenesis)	1729-1742	TTGPPPKPRRSKKA
RIMS2	RIMS2	Q9UQ26	GO:0060627 (regulation of vesicle-mediated transport)	522-535	VRPPPPKPHKSKKG

RIOK3	RIOK3	O14730	GO:1901699 (cellular response to nitrogen compound); GO:1902532 (negative regulation of intracellular signal transduction)	137-150	TRDDPYRPAKPVPT
RNF25	RNF25	Q96BH1		335-348	AMLDPPKPSRGPWR
RYBP	RYBP	Q8N488		5-18	DKKSPTRPKRQAKP
SASH1	SASH1	O94885	GO:0045859 (regulation of protein kinase activity)	616-629	DEEKPKRPTRRRRK
C11orf31	SELH	Q8IZQ5		67-80	VKVNPTKPRRGSFE
SH3D19	MTMR5	Q5HYK7	GO:0009896 (positive regulation of catabolic process); GO:0022604 (regulation of cell morphogenesis)	258-271	VIRIPAKPGKCLHE
**SH3KBP1**	**SH3K1**	**Q96B97**	**GO:0006897 (endocytosis); GO:0022604 (regulation of cell morphogenesis)**	**415-429**	**GALPPRRPERPVGP**
SHANK2	SHAN2	Q9UPX8	GO:0050808 (synapse organization); GO:1902532 (negative regulation of intracellular signal transduction)	999-1012	AVYVPAKPARRKGM
SHANK3	SHAN3	Q9BYB0		911-924	AATPPERPKRRPRP
INPP5D	SHIP1	Q92835	GO:0050852 (T cell receptor signaling pathway); GO:0008285 (negative regulation of cell population proliferation)	1117-1130	PAKRPIKPSRSEIN
SHISA6	SHSA6	Q6ZSJ9	GO:0051668 (localization within membrane); GO:0098962 (regulation of postsynaptic neurotransmitter receptor activity)	1327-1340	KAPRPAKPHKPPGS
SMG8	SMG8	Q8ND04	GO:0045859 (regulation of protein kinase activity)	284-297	DPAHPDKPKKHSPK
C9orf24	SMRP1	Q8NCR6		144-157	PLECPPRPERLNAY
SPERT	SPERT	Q8NA61		434-444	RSQDPKKPSRV
SYNJ1	SYNJ1	O43426	GO:0060627 (regulation of vesicle-mediated transport); GO:0006897 (endocytosis); GO:0051640 (organelle localization)	1061-1074	VPSLPIRPSRAPSR

TANC2	TANC2	Q9HCD6	GO:0120035 (regulation of plasma membrane bounded cell projection organization);<br>GO:0051640 (organelle localization)	1975-1988	GQTSPIKPKRPFVE
TNKS1BP1	TB182	Q9C0C2	GO:0009896 (positive regulation of catabolic process)	1698-1711	PLTLPPKPEKSSGS
TDRD3	TDRD3	Q9H7E2		529-542	RRSGPIKPEKILES
THEMIS	THMS1	Q8N1K5	GO:0050852 (T cell receptor signaling pathway)	550-563	ASHPPPRPPKHPSV
THSD7A	THS7A	Q9UPZ6		1629-1642	ACKPKKPQRRQNN
TULP1	TULP1	O00294	GO:0060627 (regulation of vesicle-mediated transport); GO:0006897 (endocytosis);<br>GO:0031175 (neuron projection development)	171-184	SPDPPPKPLRVRNK
ZCCHC6	TUT7	Q5VYS8	GO:0009896 (positive regulation of catabolic process)	178-191	QRSRPRKPRKTRNE
KIAA1429	VIR	Q69YN4		1670-1683	GIPPPKRPLKVSQK
WDR44	WDR44	Q5JSH3		207-220	EEVAPAKPPRHLTP
WDR7	WDR7	Q9Y4E6		914-927	MKKGPTRPPRPSTP
ZNF746	ZN746	Q6NUN9		344-357	GLREPARPERDMGE
ZSWIM8	ZSWM8	A7E2V4	GO:0009896 (positive regulation of catabolic process)	1127-1140	GWGSPGRPKKKHTG

The previously-reported targets recognized by ASAP/GRAF/SKAP proteins are listed in bold.
